# Screening, molecular identification, population diversity, and antibiotic susceptibility pattern of Actinomycetes species isolated from meat and meat products of slaughterhouses, restaurants, and meat stores of a developing country, Iran

**DOI:** 10.3389/fmicb.2023.1134368

**Published:** 2023-07-13

**Authors:** Tahereh Motallebirad, Omid Mardanshah, Mehdi Safarabadi, Kazem Ghaffari, Mohammad Ali Orouji, Behnam Abedi, Davood Azadi

**Affiliations:** ^1^Molecular and Medicine Research Center, Khomein University of Medical Sciences, Khomein, Iran; ^2^Department of Parasitology and Mycology, School of Medicine, Isfahan University of Medical Sciences, Isfahan, Iran; ^3^Department of Laboratory Sciences, Khomein University of Medical Sciences, Khomein, Iran

**Keywords:** Actinomycetes, meat and meat product, 16SrRNA, food born infection, DST

## Abstract

**Introduction:**

Actinomycetes can colonize surfaces of tools and equipment and can be transferred to meat and meat products during manufacture, processing, handling, and storage. Moreover, washing the meat does not eliminate the microorganisms; it only spreads them. As a result, these opportunistic pathogens can enter the human body and cause various infections. Therefore, the aim of the current study was to screen, identify, and determine the antibiotic susceptibility of Actinomycetes species from meat and meat products in the Markazi province of Iran.

**Methods:**

A total of 60 meat and meat product samples, including minced meat, mutton, beef, chicken, hamburgers, and sausages, were collected from slaughterhouses, butchers, and restaurants in the Markazi province of Iran. The samples were analyzed using standard microbiological protocols for the isolation and characterization of Actinomycetes. PCR amplification of hsp65 and 16SrRNA genes and sequence analysis of 16SrRNA were used for genus and species identification. The minimum inhibitory concentrations (MICs) of antimicrobial agents were determined by the broth microdilution method and interpreted according to the CLSI guidelines.

**Results:**

A total of 21 (35%) Actinomycetes isolates from 5 genera and 12 species were isolated from 60 samples. The most prevalent Actinomycetes were from the genus *Mycobacterium*, with six (28.6%) isolates (*M. avium complex*, *M. terrae*, *M. smegmatis*, and *M. novocastrense*), followed by the genus *Rhodococcus* with five (23.8%) isolates (*R. equi* and *R. erythropolis*), the genus Actinomyces with four (19.1%) isolates (*A. ruminicola and A. viscosus*), the genus *Nocardia* with four (19.1%) isolates (*N. asiatica*, *N. seriolae*, and *N. niigatensis*), and the genus *Streptomyces* with two (9.5%) isolates (*S. albus*). Chicken and sausage samples had the highest and lowest levels of contamination, with six and one isolates. Respectively, the results of drug susceptibility testing (DST) showed that all isolates were susceptible to Ofloxacin, Amikacin, Ciprofloxacin, and Levofloxacin, whereas all of them were resistant to Doxycycline and Rifampicin.

**Discussion:**

The findings suggest that meat and meat products play an important role as a reservoir for the transmission of Actinomycetes to humans, thus causing life-threatening foodborne diseases such as gastrointestinal and cutaneous disorders. Therefore, it is essential to incorporate basic hygiene measures into the cycle of meat production to ensure food safety.

## Introduction

In recent decades, environmental microbial pollution has become an increasingly pressing issue in food safety (Sofos, 2008). An alarming number of foodborne disease outbreaks have been reported, where animal products and fresh produce were contaminated with pathogenic protozoa, viruses, and bacteria (Machado-Moreira et al., 2019). According to the World Health Organization (WHO) 2017 report, more than 600 million illnesses, and 351,000 deaths occur annually worldwide due to food polluted with bacterial pathogens (Kirk et al., 2017). Meat and meat products are considered one of the major transmission tools for bacterial pathogens and account for over 16% of all foodborne epidemics worldwide (Tesson et al., 2020). Microbial contamination of meat and meat products occurs during preparation, processing, handling, and distribution. The safety of meat and meat products is affected by many chemical, physical, and biological hazards. However, biological hazards pose the highest foodborne risk for consumers (Gao et al., 2022).

When considering biological hazards, bacterial pathogens are of the most significant concern regarding meat and meat product safety for consumers (Gao et al., 2022). Pathogenic microorganisms, such as the Actinomycetes group (*Mycobacterium*, *Nocardia*, *Rhodococcus*, and *Streptomyces*), are members of the normal microbiota of animals and humans and are abundant in the environment. Therefore, they have a high potential for cross-contamination of meat and meat products, which can lead to foodborne diseases (Buzby, 2001; Eltholth et al., 2009). The primary outcome of food contamination is the prevalence of pathogens in the population. As a secondary consequence, antibiotic resistance is created in that society. This issue not only causes disease spread but also incurs significant economic costs to society (Capita et al., 2013).

Foodborne pathogens, such as *Salmonella* spp., *Escherichia coli*, *Campylobacter*, *Aeromonas*, *Staphylococcus aureus*, and particularly members of the Actinomycetes group (*Mycobacterium*, *Nocardia*, *Rhodococcus*, and *Streptomyces*), originate from animals and the environment during slaughtering, preparation, processing, handling, and distribution (Rouger et al., 2017). These bacteria contaminate the carcass and are transferred to cut and minced meat intended for further processing, thereby contaminating these food sources and spreading infections to consumers. Bacterial spoilage of meat and its products depends on the primary number of microorganisms, temperature and time of storage conditions, and physical and chemical properties of meat (Odeyemi et al., 2020). Contamination often occurs due to improper sanitary conditions and handling in slaughterhouses and butchers during the preparation and production process of meat products (Odeyemi et al., 2020; Tesfaye, 2021). Furthermore, the attachment and biofilm formation properties of contaminant bacteria on surfaces in slaughterhouses and butchers facilitate the cross-contamination of meat (Odeyemi et al., 2020; Tesfaye, 2021). Preslaughter conditions, such as housing and feeding, including pollution from skin and feces, contaminated water, and contents of the digestive system, are sources of diverse pathogenic bacteria such as *Staphylococcus*, *Escherichia*, *Bacillus*, and *Actinomycetes* (Bakhtiary et al., 2016). In general, to prevent the spread of such pathogenic bacteria through meat, the hygienic level and degree of contamination of centers related to meat production and preparation must be continuously monitored.

Actinomycetes form a phylogenetically coherent group that includes the *Corynebacterium, Nocardia, Rhodococcus, Arthrobacter, Streptomyces, Gordonae,* and *Mycobacterium* genera. They have one of the broadest host ranges of all known pathogens and have been reported in a variety of mammals, poultry, and reptiles (Goodfellow and Williams, 1983; Azadi et al., 2020). Additionally, Actinomycetes have high intrinsic resistance, which allows them to survive and grow in harsh environments, such as natural water and soil resources. As a result, this group of bacteria can not only cause disease directly in animals but can also contaminate meat and meat products due to their presence in environmental sources and be transmitted to humans through them (Tiwari and Gupta, 2013). For these reasons, isolating, identifying, and controlling the sources of these infections, including foods such as meat and meat products, is one of the most critical health issues worldwide.

Drug susceptibility testing (DST) results can vary significantly between Actinomycetes species. For example, some *Nocardia*, *Mycobacterium*, *Rhodococcus*, etc. are sensitive to aminoglycosides and macrolides, while other species are resistant to these drugs. Therefore, DST should be performed to determine the antibiotic susceptibility of pathogenic and opportunistic species for suitable treatment (Njeru et al., 2019).

Based on the literature, the majority of infections related to the Actinomycetes group have been reported in developed countries (Lai and Hsueh, 2014; Könönen and Wade, 2015; Azadi et al., 2020; Duggal and Chugh, 2020). However, in developing countries such as Iran, there have been no comprehensive reports on the contamination or prevalence of Actinomycetes in meat and meat products. Moreover, in developing countries, the characterization of Actinomycetes group members has been done using traditional laboratory methods, such as direct microscopy tests, including acid-fast and gram staining. Consequently, many of these microbial agents are not recognized and can be transmitted through various sources, including food (Aghamirian and Ghiasian, 2009). Therefore, the aim of the current study was to screen, identify, and evaluate the population diversity and antibiotic susceptibility pattern of Actinomycetes species in meat and meat products of the Markazi province of Iran using phenotypic and molecular methods. This study aims to provide a better insight into their role as one of the most important pathogens in the transmission and development of foodborne infections.

## Materials and methods

### Study design, sampling, and decontamination

In a cross-sectional study conducted between July 2021 and March 2022, a total of 60 meat and meat product samples were collected, including 10 mutton, 15 beef, 10 chicken, 5 minced meat, 7 sausages, 7 hot dogs, and 6 hamburgers. The samples were collected from slaughterhouses, butchers, and restaurants in the Markazi province of Iran and were transferred aseptically to the laboratory of Khomein University of Medical Sciences. The samples were processed within a maximum period of 24 h. The details of the collected samples are presented in Table 1. The number of samples was determined based on similar studies, the number of sampling centers, and the statistical formula given in the sample size section.

**Table 1:** Samples profile, phenotypic, and molecular features of Iranian Actinomycetes isolated from meat and meat product.

	Sample profile					Phenotypic features											16S rRNA analysis		
Isolates designation	Sample type	Sampling location	Tm	pH	Opt. Tm	Growth rate	Pigment production	Niacin accumulation	Nitrate reduction	Catalase	Tween 80 hydrolyses	Urease	Lysozyme resistance	Composition of tyrosine	Decomposition of xanthene	Decomposition of hypoxanthine	Similarity (%)	Base pair differences	Identification
MA1 and MA2	Chicken/beef	Restaurant/slaughterhouse	4	6	35	S	N	−	−	+	+	−	−	−	−	−	100	0/930	*M. avium complex*
MA3 and MA4	Hamburger/mutton	Restaurant	2–4	8	35	S	N	−	−	+	−	−	−	−	−	−	99.9	1/823	*M. terrae*
MA5	Minced meat	Butcher	2	7.4	35	R	Yellow	−	−	+	−	+	−	−	−	−	100	0/740	*M. smegmatis*
MA6	Chicken	Butcher	4	7.2	35	R	White	−	+	−	+	−	−	−	−	−	100	0/890	*M. novocasterense*
MA7, MA8, and MA9	Beef/minced Meat/mutton	Slaughterhouse/butcher/restaurant	2–6	6.8–7.6	30	R	Yellow	−	+	+		+	−	+	+	+	99.9	1/886	*R. equi*
MA10 and MA11	Chicken/beef	slaughterhouse/restaurant	2–4	6.3–8	30	R	White	−	+	+	+	+	−	+	+	−	100	0/680	*R. erythropolis*
MA12, MA13, and MA14	Chicken/sausage/	slaughterhouse/restaurant	2–6	7.3–7.8	30	R	White	−	−	+	−	+	−	−	+	−	100	0/890	*A. ruminicola*
MA15	Hamburger	Restaurant	2	7.6	30	R	White	−	−	+	−	+	−	−	+	−	100	0/745	*A. viscosus*
MA16 and MA17	Hot dog/minced meat	Butcher/restaurant	4	7.8	35	R	Pinkish	−	+	−	−	−	+	−	+	−	99.8	2/735	*N. asiatica*
MA18	beef	Slaughterhouse	4	7.6	30	R	Yellow	−	−	+	−	−	+	−	−	+	100	0/635	*N. seriolae*
MA19	Mutton	Butcher	6	7.2	35	R	Pink	−	−	−	+	+	+	−	+	−	100	0/812	*N. niigatensis*
MA20 and MA21	Chicken/mutton	slaughterhouse/restaurant	2–4	7.8	30	R	Red	−	−	+	−	+	−	−	−	+	100	0/887	*S. albus*

Base pair differences: the number of nucleotide differences between isolates and the nearest validated species. Tm, Temperature; R, rapid grower < 7 days; S, slow grower > 7 days; Similarity, % similarity to the nearest validated species.

Initially, a small portion of each collected sample was sliced off and minced to measure its temperature and pH. The electrode of a pH meter and thermometer was placed inside the minced meat to obtain these measurements. Then, the samples underwent processing and pretreatment based on standard protocols for the isolation and characterization of Actinomycetes species (Verdugo et al., 2014; Siavashifar et al., 2021). In brief, approximately 2–4 grams of each sample were transferred to a homogenized bag and homogenized using a laboratory blender stomacher (CARL ROTH-Karlsruhe-Germany). The suspension was then decontaminated with 3% sodium lauryl sulfate and 1% NaOH for 10 min to decrease the number of contaminants, such as Protista, fungi, and other bacteria. The tube was subsequently centrifuged at 4,000×*g* for 20 min, and 100 μL of the supernatant and pellet were cultured on blood agar, Sauton’s media [supplemented with nalidixic acid, nystatin, and kanamycin (each 50 μg/mL^−1^)], and Lowenstein Jensen (LJ) media. The cultures were incubated at temperatures of 20°C, 30°C, and 37°C in an atmosphere of 10% CO_2_ for 2 months.

### Microbiological identification of the isolates

The isolates were initially identified as Actinomycetes (*Rhodococcus*, *Nocardia*, *Streptomyces*, and *Mycobacterium*) using conventional microbiological methods, including acid-fast, partially acid-fast, and gram staining. Other tests such as CAMP test with *Listeria ivanovii* (ATCC 19119) as the indicator, pyrazinamidase, growth at 20°C, 30°C, 35°C, and 42°C, esculin, urease activity, catalase, tellurite reduction, nitrate reduction, tween opacity, niacin accumulation, resistance to lysozyme, pigment production, hydrolysis of xanthine, tyrosine, and hypoxanthine were also performed (McNeil and Brown, 1994; Saubolle and Sussland, 2003). Further identification was pursued using a panel of molecular assays as follows:

### Molecular identification

The chromosomal DNA of the Actinomycetes isolates was obtained using the method described by Azadi et al. (2020). In brief, a few colonies of bacteria grown on Sutton medium were added to 200 μL of TE buffer [1 mM EDTA, 10 mM Tris (pH 8)] and boiled for 15 min at 100°C. The microtube was then placed in a −20°C freezer for 20 min, and this procedure was repeated twice. The suspension was centrifuged at 8,000×*g* for 10 min, and the supernatant was transferred to a new microtube and centrifuged at 13,000×*g* for 20 min. The pellet was resuspended in 50 μL of TE buffer and stored at −20°C.

The isolates were initially identified phenotypically as Actinomycetes and further identified to the genus level using a genus-specific PCR based on a 596 bp region of the 16S rRNA gene, an 829 bp fragment of the 16S rRNA gene, and a 620-bp fragment of the hsp65 gene for *Streptomyces*, *Nocardia*, *Rhodococcus*, and *Mycobacterium*, respectively (Laurent et al., 1999; Khan and Yadav, 2004; Shojaei et al., 2011). PCR amplification and sequence analysis of the almost complete 16S rRNA gene were used for species identification of the isolates, as described by Azadi et al. Sequencing was performed at Pishgam Biotech Company (Iran) (Azadi et al., 2020). The obtained sequences were manually aligned and compared and analyzed with all sequences of closely related Actinomycetes species retrieved from the GenBank database using the jPhydit program version 1.1.3 (Jeon et al., 2005).

### Drug susceptibility testing (DST)

For all isolated Actinomycetes, DST was performed using the broth microdilution method according to the Clinical and Laboratory Standards Institute (CLSI) 2021 guidelines (CLSI, 2021). Cefoxitin, amikacin, doxycycline, ciprofloxacin, rifampicin, kanamycin, isoniazid, streptomycin, levofloxacin, and imipenem were selected from each antibiotic class introduced in CLSI for DST. Since meat can be a source of various infections in humans, one antibiotic was chosen from each antibiotic class for testing. Stock solutions of each antibiotic were prepared by dissolving the powder of each antibiotic in a suitable solvent, and two-fold serial dilutions for all antibiotics ranging from 0.06 to 512 mg/L were prepared into 96-well microtiter plates. All isolated Actinomycetes were cultured in Sauton’s media and incubated at 35°C for 4 days. Then, 0.5 McFarland turbidity was prepared for each isolate by dissolving the colonies grown on Sauton’s medium in the broth. Afterward, 0.1 mL of this turbidity was inoculated into each dilution and incubated at 37°C. The lowest concentration of the antibiotic that inhibited bacterial growth was determined as the (MIC). *Mycobacterium peregrinum* ATCC 700686, *Enterococcus faecalis* ATCC 29212, and *Staphylococcus aureus* were used as quality controls of DST (Woods et al., 2011).

#### Nucleotide sequence accession numbers

The GenBank accession numbers for the 16S rRNA sequencing of isolated Actinomycetes in this study are listed below: isolate MA1 *M. avium* complex (OP520901), isolate MA3 *M. terrae* (OP520902), isolate MA5 *M. smegmatis* (OP520903), isolate MA7 *R. equi* (OP520904), isolate MA6 *M. novocastrense* (OP520905), isolate MA10 *R. erythropolis* (OP520907), isolate MA12 *A. ruminicola* (OP520908), isolate MA15 *A. viscosus* (OP520909), isolate MA16 *N. asiatica* (OP520910), isolate MA18 *N. seriolae* (OP520912), isolate MA19 *N. niigatensis* (OP520915), and isolate MA20 *S. albus* (OP520914).

### Sample size

According to the literature, the prevalence of Actinomycetes in meat and meat products is 20 (Lai and Hsueh, 2014; Könönen and Wade, 2015; Azadi et al., 2020; Duggal and Chugh, 2020). To detect the minimum sample size, the formula used was:


$$n=\frac{z^{2}\hat{p}\left(1-\hat{p}\right)}{d^{2}}$$

where, *n* = sample size, *z* = statistics corresponding to a 95% level of confidence, $\hat{p}$ = expected prevalence (5%), and *d* = precision (5%). On the basis of this formula, the sample size required was 60.

### Statistical analysis

The tests were conducted in duplicates, and the average values were calculated for the final results. The prevalence of Actinomycetes isolates in the tested samples was calculated using the following formula: (number of positive samples/total sample size) × 100. The results were then expressed as a percentage. The data generated in this study were presented in tables and percentages.

## Result

The recorded pH and temperature of the meat and meat product samples were between 6–8.5°C and 2–8°C, respectively. In the current study, a total number of 21(35%) Actinomycetes isolates were recovered and characterized from 60 meat and meat product samples including mutton, beef, chicken, minced meat, sausages, hot dogs, and hamburgers which were collected from slaughterhouses, butchers, and restaurants of the Markazi province of Iran. A total of 16 (26.66%) of 60 samples were positive for Actinomycetes. A total of 14 (35%) of 40 sampling sites were positive for Actinomycetes, while in 26 other sampling sites, no Actinomycetes species were detected. Among all isolates, six (28.6%) isolates were recovered from chicken, four (19%) isolates were recovered from mutton samples, four (19%) isolates were recovered from beef, three (14.28%) isolates were recovered from minced meat, two (9.52%) isolates were recovered from hamburgers, one (4.76%) isolate was recovered from sausages, and one (4.76%) isolate was recovered from hot dogs. A total number of 24 (40%) samples were contaminated with fungi, gram-positive, and gram-negative bacteria. The details of meat and meat product samples and the Actinomycetes isolates are shown in Table 1.

Based on culture, biochemical and phenotypical features, and the genus-specific PCR, including the presence of a 596 bp fragment of the 16SrRNA and a 618-bp fragment of the hsp65, all 21 isolates were characterized as Actinomycetes (*Rhodococcus*, *Nocardia*, *Mycobacterium*, *Streptomyces*, and *Actinomyces*). Of these 21 isolates, six isolates were identified as *Mycobacterium*, five isolates were identified as *Rhodococcus*, four isolates were identified as *Actinomyces*, four isolates were identified as *Nocardia*, and two isolates were identified as *Streptomyces*.

The complete 16SrRNA gene sequences analysis of isolates showed that all our isolates had nucleotide signatures of Actinomycetes at positions 70–98 (A-T), 293–304 (G-T), 307 (C), 328 (T), 614–626 (A-T), 631(G), 661–744 (G-C), 825–875 (A-T), 824–876 (T-A), 843 (C), and 1,122–1,151 (A-T) for *Streptomyces*, *Nocardia*, and *Rhodococcus* and at positions 70–98 (U-A), 139–224 (G-C), 843 (C), 1,189 (C), 1,244–129 (C-G), 1,308–1,329 (C-G), and 1,008–1,021 (C-G) for *Mycobacterium* sp.

The isolates belonged to 5 genera and 12 validated species. The most prevalent Actinomycetes in this study were genus *Mycobacterium* with six (28.6%) isolates, which includes *M. avium* complex with two isolates (MA1 and MA2), *M. terrae* with two isolates (MA3 and MA4), *M. smegmatis* with one isolate (MA5), and *M. novocastrense* (MA6). This is followed by genus *Rhodococcus* with five (23.8) isolates including *R. equi with three isolates* (MA7, MA8, and MA9) and *R. erythropolis* with two isolates (MA10 and MA11), genus *Actinomyces* with four (19.1) isolates including *A. ruminicola* with three isolates (MA12, MA13, and MA14) and *A. viscosus* with one isolate (MA15), genus *Nocardia* with four (19.1) isolates including *N. asiatica* with two isolates (MA16 and MA17), *N. seriolae* with one isolate (MA18), and *N. niigatensis* with one isolate (MA19), and genus *Streptomyces* with two (9.5%) isolates including *S. albus* (MA20 and MA21).

**Figure 1:**
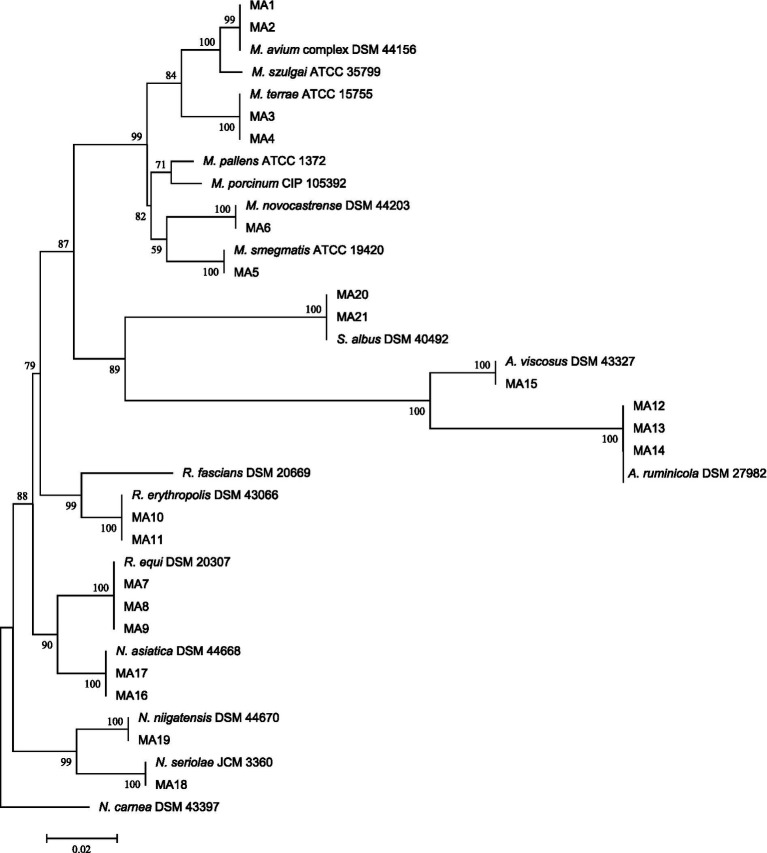
16S rRNA sequence-based phylogenetic tree for *Actinomycetes* species and nearest standard species of *Actinomycetes* by using the neighbor-joining method depicted by MEGA8 software. At each node, bootstrapping values are represented.

The relationship between our Actinomycetes isolates and validated established Actinomycetes species was depicted using a high bootstrap value phylogenetic tree of the 16S rRNA gene by MEGA 8 software. The neighbor-joining method with an arithmetic mean of pairwise differences matrix was used (Figure 1).

### DST results

Table 2 presents the MIC of 10 selected antibiotics against 21 Actinomycetes isolates from meat and meat products. The lowest MIC for selected antibiotic agents against our isolates belonged to ciprofloxacin, levofloxacin, and ofloxacin, with a range of 0.25–2 μg/mL, while the highest MIC of antibiotic agents belonged to isoniazid and doxycycline, with a range of 4 μg/mL. Actinomycetes species isolated from beef and mutton had a higher MIC for imipenem, doxycycline, rifampicin, and streptomycin, while they had a low MIC value for cefoxitin, ofloxacin, and ciprofloxacin. Actinomycetes species isolated from chicken had a high MIC for doxycycline, cefoxitin, and amikacin, while they had a low MIC for kanamycin, streptomycin, and levofloxacin.

**Table 2:** Average MIC of Actinomycete isolates to selected antibiotics according to CLSI 2021 standard.

isolates	Species	Rifampicin	Isoniazid	Streptomycin	Amikacin	Kanamycin	Ciprofloxacin	imipenem	Levofloxacin	Cefoxitin	Doxycycline
MA1 and MA2	*M. avium complex*	≥128(R)	≥256(R)	≥256(R)	4(S)	4(S)	≤0.5(S)	≤0.25(S)	≤0.5(S)	32(I)	≥256(R)
MA3 and MA4	*M. terrae*	≥64(I)	≥256(R)	≥128(R)	8(I)	6(S)	≤0.5(S)	≤0.25(S)	≤0.5(S)	32(I)	≥256(R)
MA5	*M. smegmatis*	≥128(R)	≥256(R)	≥128(R)	4(S)	4(S)	≤0.25(S)	≤0.5(S)	≤0.5(S)	32(I)	≥256(R)
MA6	*M. novocastrense*	≥256(R)	≥128(R)	≥256(R)	4(S)	4(S)	≤0.5(S)	≤0.5(S)	≤0.5(S)	64(R)	≥128(R)
MA7, MA8, and MA9	*R. equi*	≤0.5	≥256(R)	2(S)	2(S)	8(I)	1(S)	2(S)	≤0.5(S)	≥256(R)	4(S)
MA10 and MA11	*R. erythropolis*	≥64(I)	≥256(R)	≥128(R)	8(I)	4(S)	≤0.5(S)	≤0.25(S)	≤0.5(S)	64(R)	≥128(R)
MA12, MA13, and MA14	*A. ruminicola*	≥128(R)	≥256(R)	≥128(R)	8(I)	4(S)	≤0.25(S)	≤0.5(S)	≤0.5(S)	32(I)	≥128(R)
MA15	*A. viscosus*	≥128(R)	≥256(R)	≥128(R)	4(S)	4(S)	≤0.5(S)	≤0.25(S)	≤0.5(S)	32(I)	≥256(R)
MA16 and MA17	*N. asiatica*	≤0.5	≥256(R)	2(S)	2(S)	8(I)	1(S)	2(S)	≤0.5(S)	≥256(R)	4(S)
MA18	*N. seriolae*	≤0.5	≥128(R)	1(S)	2(S)	8(I)	1(S)	2(S)	≤0.5(S)	≥256(R)	2(S)
MA19	*N. niigatensis*	≥ 64(I)	≤ 1(S)	≤ 3(S)	≥ 128(R)	≤ 1(S)	≤ 1	≥ 32(R)	≤ 2(S)	≤ 1(S)	4(S)
MA20 and MA21	*S. albus*	≥64(I)	≥256(R)	≥128(R)	2(S)	4(S)	≤0.5	≤0.25(S)	≤0.5(S)	32(I)	≥256(R)

R, Resistant; I, Intermediate; S, Susceptible.

## Discussion

The Actinomycetes group is widely distributed in nature and abundantly found as members in the normal microflora of the gastrointestinal tract, oropharynx, genital tract, and upper respiratory tract of various animals and poultry (Devanshi et al., 2021; Latha and Dhanasekaran, 2021; Pavlik et al., 2022). These environmental microorganisms can colonize the surfaces of tools and equipment and be transferred to meat and meat products. Likewise, microflora can also enter food through manufacturing, processing, handling, and storage (Tiwari and Gupta, 2013; Zwirzitz et al., 2020). Moreover, although disinfection and cleaning procedures are conventionally used in food industries, they are not always effective in eradicating the resident microorganisms within each product (Cappitelli et al., 2014). Therefore, these opportunistic pathogens can enter the human body and cause various infections, including cutaneous, gastrointestinal, and respiratory infections (Cappitelli et al., 2014).

In recent decades, members of the Actinomycetes group, such as Mycobacterium, Streptomyces, Nocardia, and Rhodococcus, have been recognized and introduced as important human and animal pathogens (Conville and Witebsky, 2015; Anandan et al., 2016). It is challenging to distinguish infections caused by the Actinomycetes group from other similar infections by conventional laboratory methods. Consequently, physicians may neglect to consider the possibility of these infections in patients (Ng et al., 2012; Hashemzadeh et al., 2021). This issue highlights the importance of accurate and timely identification of Actinomycetes infections to ensure appropriate treatment. Advanced molecular techniques, such as PCR and DNA sequencing, have provided valuable tools for the accurate identification of Actinomycetes infections in recent years (Cappitelli et al., 2014).

Isolating and characterizing the entire spectrum of Actinomycetes members from meat and meat product samples is difficult and often requires specific procedures. Identifying this group requires at least 10 phenotypic and biochemical tests (Gajdács et al., 2017). Therefore, attempting to identify Actinomycetes group members only by routine conventional tests may lead to misidentification or failure to recognize similar species (Maukonen et al., 2003; Siavashifar et al., 2021). To overcome this problem and for more accurate and rapid characterization of Actinomycetes, the appropriate use of phenotypic tests, in conjunction with molecular tests, has shown promising results (Woo et al., 2008; Azadi et al., 2020). Due to this issue and also given the increasing reports of Actinomycetes group isolation from the environment, meat, meat products, and clinical samples, we conducted this study to determine the diversity of Actinomycetes in meat and meat products of the Markazi province of Iran using conventional and molecular microbiological methods. This study aims to provide a better understanding of the prevalence and potential risks of Actinomycetes in meat and meat products and to contribute to efforts to prevent and control infections caused by these microorganisms.

In this study, according to the mentioned statistical formula, the number of sampling locations, and the number of samples in similar studies, 60 meat and meat products samples were collected, of which 21 (35%) actinomycete isolates were isolated. This isolation rate is consistent with other actinomycete isolation reports (Cansian et al., 2005; Ibrahim et al., 2014; Trimoulinard et al., 2017). However, this result cannot be accurately reported as the prevalence rate due to the limitation and small volume of samples. Therefore, it is recommended that in future studies, the sample size should be increased to determine the exact prevalence of Actinomycetes. This will provide a more accurate understanding of the potential risks of Actinomycetes in meat and meat products and contribute to efforts to prevent and control infections caused by these microorganisms.

In the current study, we recovered 21 (35%) Actinomycetes isolates from 60 meat and meat product samples, including six (28.6%) isolates from chicken, four (19%) isolates from mutton, four (19%) isolates from beef, three (14.28%) isolates from minced meat, two (9.52%) isolates from hamburgers, one (4.76%) isolate from sausage, and one (4.76%) isolate from hot dogs. Since there were no published reports on the isolation of Actinomycetes species from meat and meat products, we were unable to compare our isolation rate with other studies.

Moreover, our results showed that chicken had the highest level of contamination, while packaged products such as sausages and hot dogs had the lowest level of contamination. These findings are consistent with other reports in which the highest amount of microbial contamination was found in chicken and the lowest in sausage, respectively (Cansian et al., 2005; Ibrahim et al., 2014; Trimoulinard et al., 2017). These results suggest that chicken may be a potential source of Actinomycetes contamination in meat and meat products and highlight the importance of implementing appropriate measures to ensure the safety of meat and meat products for consumers.

The Actinomycetes species isolated from meat and meat product samples in this study belonged to 5 genera and 12 species including, four *Mycobacterium* species consisting of *M. avium* complex, *M. novocastrense*, *M. terrae*, and *M. smegmatis;* three *Nocardia* species including *N. asiatica*, *N. seriolae*, and *N. niigatensis;* two *Rhodococcus* species including *R. erythropolis* and *R. equi*; two *Actinomyces* species including *A. ruminicola* and *A. viscosus*; and a species of *Streptomyces* named *S. albus.* The results of our study in accordance with other studies showed that the *M. avium* complex, *R. equi*, and *N. niigatensis* are the most prevalent species isolated from meat and meat products of the Markazi province and other parts of the world (Ghabeli Zaherkandi et al., 2021; Żychska et al., 2021).

Furthermore, based on the pathogenicity literature of Actinomycetes, it was determined that our isolates included eight opportunist pathogen species including *M. avium* complex, *M. novocastrense*, *M. terrae*, *N. asiatica*, *M. smegmatis*, *R. equi*, *A. ruminicola*, and *N. niigatensis* and four saprophyte or non-pathogen species (Savini et al., 2012; Conville and Witebsky, 2015). Actinomycetes species are zoonotic and can be transmitted to humans through the ingestion of animal-origin food, including meat and meat products (Javed et al., 2014; Weese, 2017). However, based on the literature, there are only a few reports that have investigated the presence of Actinomycetes in meat and meat products, where Actinomycetes species such as *Mycobacterium, Nocardia, Rhodococcus,* and *Streptomyces* have been isolated from pork, beef, lamb, and other sources (Lorencova et al., 2014; Portilho et al., 2019). This issue highlights the potential risk of meat and meat products as a source of transmission of these opportunistic infections to humans. Therefore, it is essential to implement appropriate measures to ensure the safety of meat and meat products and prevent the transmission of Actinomycetes infections to consumers. This includes proper handling, storage, and cooking of meat and meat products, as well as implementing effective sanitation and hygiene.

Infections caused by Actinomycetes are transmitted to humans from various sources, including water and food. Moreover, due to the use of traditional methods for the identification of Actinomycetes in developing countries, which may lead to misdiagnosis with mycosis, mycetoma, and tuberculosis, accurate molecular identification and DST are necessary (Azadi et al., 2020; Pan et al., 2021). Hence, the determination of DST of Actinomycetes isolated from food is an important decision tool that may be useful in the management and treatment of patients who are infected in this way. Therefore, in this research, for all Actinomycetes isolated from meat and meat products, DST was performed based on the CLSI 2021 standard.

Considering that the aim of this study was to find the source of transmission of Actinomycetes infection that causes diseases in humans, the selection of antibiotics was based on the CLSI standard and the type of disease that can be caused in humans, as well as the presence of all antibiotic families. However, given that these bacteria were isolated from animals, it would be better to investigate the sensitivity of Actinomycetes against antibiotics used in the livestock and poultry industry. This will provide a better understanding of the potential risks of antibiotic resistance in Actinomycetes and contribute to efforts to prevent and control infections caused by these microorganisms.

The results of DST of Actinomycetes in this study showed that all isolates from beef and mutton had higher MIC values for Imipenem, Doxycycline, Rifampicin, and Streptomycin, while they had lower MIC values for Cefoxitin, Ofloxacin, and Ciprofloxacin. On the other hand, all species isolated from chicken had higher MIC values for Doxycycline, Cefoxitin, and Amikacin, while they had lower MIC values for Kanamycin, Streptomycin, and Levofloxacin. These results are consistent with other studies that have shown that Actinomycetes have high resistance to different antibiotics and can infect humans through food sources. The high resistance of Actinomycetes to various antibiotics poses a significant challenge in the management and treatment of infections caused by these microorganisms. Therefore, it is crucial to implement appropriate measures to prevent and control the spread of antibiotic resistance in Actinomycetes and to use antibiotics judiciously in both human and animal health to minimize the development and spread of antibiotic resistance. Additionally, further studies are needed to investigate the prevalence of antibiotic resistance in Actinomycetes isolated from different sources, including livestock and poultry, to better understand the potential risks of antibiotic resistance in these microorganisms and to inform appropriate control strategies (Capasso, 2007; Waddell et al., 2016).

In this study, a total of six non-tuberculosis *Mycobacterium* (NTM) isolates were tested for antimicrobial susceptibility, and the results showed that 100% of isolates were resistant to rifampicin, isoniazid, streptomycin, and doxycycline, while around 25% of isolates were resistant to kanamycin, cefoxitin, and amikacin. Moreover, 100% of the isolate was susceptible to ciprofloxacin, imipenem, and levofloxacin. Additionally, a total of six Nocardia isolates were tested for antimicrobial susceptibility, and the results showed that 80% of isolates had resistance to isoniazid, kanamycin, and cefoxitin, while around 90% of them were susceptible to other tested antibiotics. These results confirm other studies that have reported high antibiotic resistance in NTM and Nocardia isolated from human, animal, and environmental resources (Azadi et al., 2016, 2020; Wang et al., 2022).

These results highlight the potential role of food, food production, and environmental sources as a vehicle for antibiotic-resistant bacteria and antibiotic-resistance genes to humans, which can have a significant public health impact. This phenomenon can induce resistance to many antibiotics, including those that are last-resort treatments for patients infected with multidrug-resistant bacteria. Therefore, it is essential to implement appropriate measures to address the issue of antibiotic-resistant food. These include information, surveillance, monitoring, education and training, record-keeping, reduction of infection, optimization and reduced antibiotic use, legislation, and sustainable investment for alternatives. These actions are important to bring antibiotic-resistant food under control and reduce the risk of antibiotic resistance in humans (Azadi et al., 2020; Pan et al., 2021).

## Conclusion

In conclusion, the results of our study show that meat and meat products from slaughterhouses, butchers, and restaurants are contaminated with high levels of bacterial pathogens such as the Actinomycetes group species. This finding highlights the significant role of meat and meat products as a reservoir for the transmission of these bacteria to humans, causing life-threatening food-borne diseases such as gastrointestinal and cutaneous disorders. Therefore, it is essential to incorporate basic hygiene measures in slaughterhouses, retail meat outlets, and restaurants to ensure food safety. Additionally, staff involved in the preparation, packaging, distribution, and cooking of meat and meat products should receive proper training on inspection procedures and food safety practices to minimize the contamination rate of raw meat and meat products offered in the market. Furthermore, the determination of DST of Actinomycetes species isolated from meat and meat products may be useful in the management of patients who are infected in this way. This will provide information on the susceptibility of these microorganisms to different antibiotics, which can inform appropriate treatment strategies and prevent the spread of antibiotic resistance. Overall, this study highlights the importance of ensuring food safety and implementing appropriate measures to prevent the transmission of bacterial pathogens, including Actinomycetes, from meat and meat products to humans.

## Data availability statement

The datasets presented in this study can be found in online repositories. The names of the repository/repositories and accession number(s) can be found in the article/supplementary material.

## Ethics statement

This study was approved by the Research Ethics Committee of Khomein University of Medical Sciences (Ethical code: IR.KHOMEIN.REC.1399.003). The authors certify that all data collected during the study are as presented in this paper, and no data from the study has been or will be published elsewhere separately.

## Author contributions

TM, OM, MS, KG, MO, BA, and DA contributed to the study’s conception and design. Material preparation, data collection, and analysis were performed by DA, OM, KG, MS, MO, and TM. The first draft of the manuscript was written by TM, DA, and KG, and MS commented on previous versions of the manuscript. All authors contributed to the article and approved the submitted version.

## Funding

This study was funded by Khomein University of Medical Sciences (Grant no. 98000003).

## Conflict of interest

The authors declare that the research was conducted in the absence of any commercial or financial relationships that could be construed as a potential conflict of interest.

## Publisher’s note

All claims expressed in this article are solely those of the authors and do not necessarily represent those of their affiliated organizations, or those of the publisher, the editors and the reviewers. Any product that may be evaluated in this article, or claim that may be made by its manufacturer, is not guaranteed or endorsed by the publisher.

## Abbreviations

WHO: World Health Organization
LJ: Lowenstein Jensen
PCR: Polymerase Chain Reaction
CAMP: Christie–Atkins–Munch-Peterson
